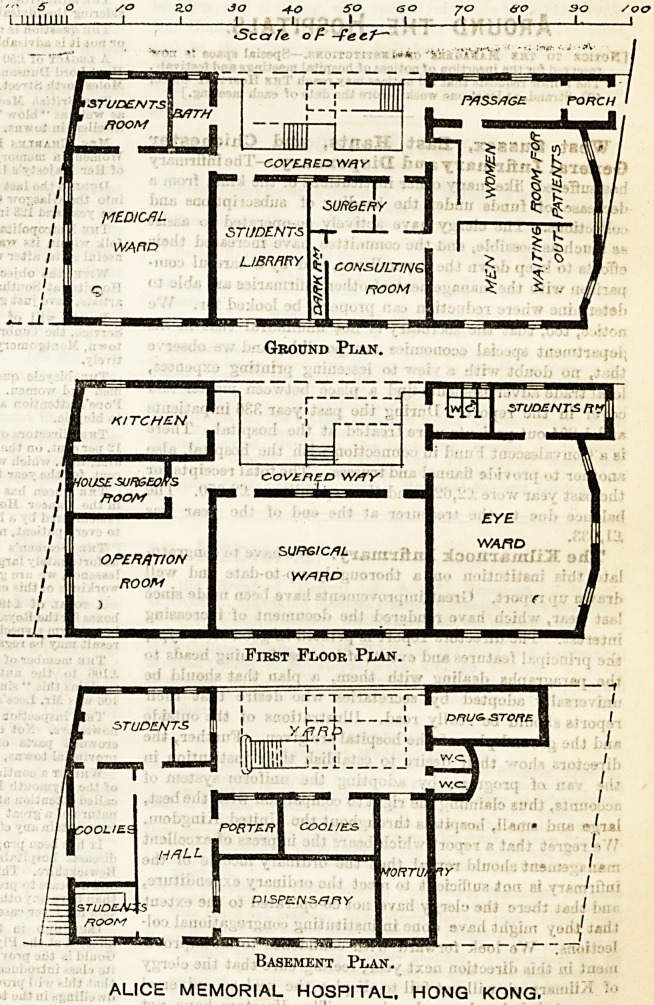# The Alice Memorial and the Nethersole Hospitals, Hong Kong

**Published:** 1895-02-02

**Authors:** 


### HOSPITAL CONSTRUCTION.

### THE ALICE MEMORIAL AND THE NETHERSOLE HOSPITALS, HONG KONG.

These are two distinct but affiliated institutions, both managed and controlled by the London Missionary Society. The Alice Memorial Hospital was founded by Dr. Ho Kai, in memory of his wife, "as a chapel for Christian worship and a hospital for healing, and the promotion of medical science" (extract from trust deed). The Nethersole Hospital was erected in 1893 by Mr. H. W. Davis, on a site belonging to the London Missionary Society, granted for the purpose, and at a cost of £2,000.

The Alice Memorial Hospital is a small building of three floors in height. In the basement are two rooms for students, two coolies' rooms, a large hall, dispensary, porters' room, and a drug store. Entered out of an open courtyard are two water-closets and the mortuary. On the ground floor to the left of the entrance passage are two waiting-rooms for out-patients, a consulting room, with surgery and dark room attached; a covered way leads to the students' library, a medical ward, out of which is a students' room, and a bath-room. The staircase is apparently in the open air. On the upper floor is a large eye ward, a surgical ward, an operation room, house surgeon's room, and kitchen; and entered from the eye ward is a students' room; the water-closets are approached from the covered way, which affords access to all these rooms.

All the patients in the wards of this hospital are men, the women's wards being in the Nethersole Hospital. The eye ward contains nineteen beds, the surgical ward nineteen, and the medical ward fifteen beds. The floor area per patient appears remarkably small according to European ideas; in the larger wards it is about fifty feet, and in the smaller forty-six feet. There does not appear to be any accommodation for the resident staff, other than the one room for the house surgeon.

The Nethersole Hospital is a long, narrow building, the first floor of which is greater in extent than the ground floor, an arrangement probably brought about by the contour of the ground.

The ground floor contains a waiting hall, with dispensary, consulting room and surgery, where female patients only are seen twice a week. On this floor are also three students' rooms, a room for the house surgeon, stores, and the mortuary. On the upper floor are two wards for men, one of twelve beds for surgical patients, the other, of seven beds, for medical patients, an isolation ward, two large wards for women, one of twelve beds for "general" patients (*i.e.*, surgical and medical?), and one of nine beds for ophthalmic patients; a small obstetric ward. matron's room, entered out of the women's general ward, and the obstetric ward; a nurses' room, formed by enclosing a part of the women's ophthalmic ward; an operation room, communicating directly with the women's general ward and the male surgical ward, two bath-rooms, water-closets, a cook's room, and the kitchen. The wards all open on to a verandah, which affords access also to the water-closets, bath-rooms, and kitchen.

ALICE MEMORIAL HOSPITAL, HONG KONG.

It seems difficult to imagine any valid reason for so extraordinary an arrangement as the direct communication between the operation theatre and the wards, or for the position of the matron's and nurses' rooms in relation to the wards. We should have thought, too, that the staircase could have been kept entirely outside the building, as it is in the Alice Memorial Hospital, instead of forming, as it must, a shaft for the conveyance of air from the ground floor to the operation-room.

The number of beds in the Nethersole Hospital is twenty-one women and nineteen men, the two hospitals together affording accommodation for ninety beds in all.

An integral part of the work done in this institution is the religious teaching which is carried on by officers of the London Missionary Society, assisted by native missionaries.

## Figures and Tables

**Figure f1:**